# The Extinction of Tuberculosis1Read before the Section on Public Health of the New York Academy of Medicine, Nov. 8, 1895.

**Published:** 1895-12

**Authors:** James Law

**Affiliations:** Cornell University, Ithaca, N. Y.


### THE EXTINCTION OF TUBERCULOSIS.[1]

By PROF. JAMES LAW,
CORNELL UNIVERSITY, ITHACA, N. Y.

In dealing with diseases in the lower animals there are various possibilities and limitations which do not enter the field of medicine as applied to man. These must ever be borne in mind if the veterinary sanitarian would have his work prove a success. As applied to the contagious disease before us this evening these considerations must largely influence us in the choice of methods and in their application in the sanitary field. Prominent among such considerations are:

I. The facility with which contagion may be checked by the death of the infected animal.

II. The possibility of arresting the career of contagion by breeding only from insusceptible strains of blood.

III. The impossibility of applying effective methods because of the economic ruin which would be entailed.

1. *Destruction of the infceted.* Under the first head, the checking of contagion by the killing of the infected, the principle being altogether inapplicable in dealing with man, the physician naturally thinks first of segregation, disinfection, and a police-control of the sick. With the veterinarian, on the other hand, free from any sense of the sacredness of the life of the sick, the first thought is a purely economical one, namely: Will the cost of the seclusion and care of this animal and of the probable infection of others outvalue the prospect of its recovery and the actual worth of the subject when well again? With any virulent and dangerous disease there can only be one answer to this question, namely: That economy demands the extinction of the contagion at the expense of the first victim before ten or a hundred more shall contract the disease. With a subject, the actual sound-value of which may have been anywhere between ten dollars and one hundred dollars, the administration of such police-control as would guarantee the prevention of extension of infection, through man, beast, bird, or inanimate object, would, as a rule, more than use up the price of the first

[1] Read before the Section on Public Health of the New York Academy of Medicine, Nov. 8, 1895.

victim. Every day of delay in shutting up these living factories of contagion places a greater balance on the wrong side. The economical soundness of this position has been now so often illustrated on a large scale and in regard to so many different diseases, as rinderpest, lung-plague, sheep-pox, glanders, and rabies, that for those who had made a special study of it, it has passed from the field of discussion into that of established truths.

2. *Insusceptible families.* In regard to the second question, of propagating insusceptible families only, while it is, equally with our first proposition, impossible of adoption in the human family, as interfering with personal rights, it is certainly available for the lower animals, over which the State can exercise the right of eminent domain and employ a police-control for the common good. In animals, as in man, there are certain families which show a strong susceptibility to given infections, and others that show an equally potent resistance, and, if it can be shown that no more serious interest is threatened by such a course, it is quite within the power of the State to exclude the first-named class from reproduction, and to breed only the families which show the comparative immunity. A principle of this kind is already in force in certain countries in which all stallions are licensed, and those calculated to deteriorate the breed of the district are excluded from stud uses. The same principle is in force in the Channel Islands, where no cattle except those of the native breed are ever allowed to enter a herd.

3. *Economic limitations.* In the third place we are confronted by the question of economic limitations. It may be said that where human health and life are involved, no question of mere economy should be allowed to enter. But the economic question may be so far-reaching that the evil to humanity would be greater in the end, and our boasted liberality for sanitary ends finally would defeat the much-coveted sanitary object. Deferring for a moment the question of the immediate influence on human health, let us glance at the national wealth in farm animals. The United States owns in round numbers $1,500,000,000 worth of live-stock, and in this are included 16,000,000 head of milch-cows and 20,000,000 head of other cattle. The cows produce 5,209,125,567 gallons of milk yearly, or over 315 gallons per head. Of the other cattle probably 5,000,000 are killed yearly for human food, representing, at least, 2,500,000,000

pounds of beef. This cannot be materially restricted without dealing a serious blow to our national prosperity.

While it must be conceded that animals would acquire a greater power of resistance to tuberculosis if kept in the open air and in a condition more nearly approximating the natural one, yet if we let the milch-cow revert to the condition of the native Texas cow, which can only scantily support its calf, and if we remand the beef-animal to its native state in which it took four years to reach maturity, in place of putting him on the market as prime beef at two years of age, we render the whole cattle-industry unprofitable. Under such conditions our stock-owners could no longer compete successfully in the markets of the world, and this branch of industry must be abandoned. But if abandoned the exhaustion of our soils is a necessary consequence, and in due time agriculture in its turn must become unprofitable, and with its decay we will reach the end of our whole national prosperity, built on our abundant agricultural products.

Whatever we do with our domestic animals we dare not cause them to revert to a state of nature. This would be to throw away the achievement of the labor and skill of centuries, and would entail the most serious retrogession of the nineteenth century civilization. As well abandon the modern triumphs in steam and electrical engineering.

The abnormal productiveness of the present farm-animal is one of the most substantial foundations of modern prosperity, and it would be an economical blunder, and finally a sanitary one, to seek its abandonment or limitation. So far as we can secure animal immunity within the productive families our work will be sound, and so far as we can produce an acquired immunity by hygienic measures we are laboring in the right line, but the line must be drawn wherever these productive measures tend to impair seriously the capacity of the breed for profitable uses.

These considerations serve to clear up our field of veterinary sanitary practice, and to narrow it to the limits that are at once the most indispensable and the most effective. One measure stands out above all others as the one which must never be lost sight of, and to which all others must be held as accessory and subservient. This measure is the extinction of the disease-germ.

*Feeding from separate troughs.* When from any cause the immediate extinction of the germ is impracticable, an all-important precaution is to teach each animal to use its own stall only, and to have stalls so separated in front that no two cattle can feed from the same trough. For the same reason no common feeding-trough should be used in yard or field, and there may be danger in using a common drinking-trough or bucket.

*Salting troughs and front of stalls.* In view of the fact that tuberculosis is largely propagated by the dried-up virulent expectorations which are raised and diffused in the form of dust, the daily sprinkling with a strong brine of the front of the stall, manger, and passage in front proves an important protective measure. Condensing the moisture of the air, the salt keeps the surface constantly damp and prevents the sputa drying up and rising as an impalpable powder. The salt is not only harmless but wholesome, and has besides a very slight bactericidal action.

*Open-air life.* So far as possible a free, open-air life should be secured for the stock. In city and suburban stables the infected may reach from 6 to 90 per cent., whereas among 2,250,000 of our fat range-cattle the tuberculous did not exceed 0.02 per cent. The comparative absence of viable bacteria in the air of the pastures, and the action of full sunshine in devitalizing the bacillus tuberculosis in a few hours, while it will live for months in an ordinary room, are sufficiently instructive in this connection. In the Northern States, where cattle must be sheltered indoors for five or six months of the year, too great care cannot be taken to secure ample windows and sunshine as well as pure air in the stables. Darkness in the stalls reduces the numbers and quality of red blood-corpuscles and the vitality and power of resistance of the animals, so that it virtually burns the candle at both ends, preserving and multiplying the germs and increasing the susceptibility of the animal.

These considerations strongly condemn the city and suburban dairy. Other things being equal, the larger the city the greater the number of floating bacilli and the greater the susceptibility of the animal system. In French towns of 5000 inhabitants the ratio of tuberculous persons is only half as much as in towns of 100,000 to 500,000. In German abattoirs the ratio of tuberculous cattle is about 5 per cent. of all slaughtered, yet Ostertag gives the ratio for old cows in the Berlin slaughter-houses as 75 per cent. The ratio is greater in our city and suburban dairies to-day than it was years ago, when a large proportion of the fresh cows were killed off by lungplague within three months of their purchase. There remains, however, this redeeming element: that the city dairyman finds it unprofitable to keep the same cows from year to year, and, as a slow disease like tuberculosis finds insufficient time for its abundant development, the result is better for the city dairy than it otherwise would be. The case is worse with a thoroughbred herd kept in the city or suburbs so long as the cows will breed, as there are here the combined evils of a generally contaminated atmosphere of indoor life in a too often infected barn, and in too many cases a bodily constitution rendered increasingly susceptible by inbreeding. Once start the infection under such conditions and the affection is liable to become concentrated by a series of successive reinfections in the same animal, so that not only are cases multiplied, but generalized cases soon become the rule rather than the exception. Everything considered, the city and suburban dairies are surrounded by greater dangers than the country ones, and this should be taken into account alike by the dairyman himself and by the sanitarian who would protect humanity against the bovine source of infection.

Among the further precautions which are within the control of the stockowner himself may be named the following:

*Avoid breeding too young.* The immature system is easily debilitated.

*Do not unduly stimulate the milk-secretion.* Let the diet be always sufficient to sustain the vital powers as well as the lactation.

*When the cow goes dry do not allow her to suffer from insufficiency or unsuitability of food.*

*Correct all conditions of ill-health.* Debility is an urgent invitation to the bacillus. On the other hand, the most phenomenal powers of digestion, assimilation, rapid growth, and fattening give no guarantee of protection when the germ is implanted. Professor Bang tells us that the disease was virtually unknown in Danish cattle until 1840, when it was introduced in some purchases from Schleswig, and later in 1850 by large importations of English short-horns, and now the Copenhagen abattoirs show 17 per cent. tuberculous, and the tuberculin-test on 19,462 cattle showed 61.6 per cent. tuberculous.

*Old or unthrifty cows should be excluded from the herd.* Twice to six times as many old cows as of young are tuberculous. The first sign of unthriftiness should be a warrant for separation from the rest of the herd, and especially in the case of the aged. Better still to use the tuberculin-test to decide whether it should be promptly slaughtered.

*Do not buy from a herd which has furnished cases of tuberculosis in recent years.* Here again the safe course is to admit only such as have stood the test with tuberculin.

*Let no tuberculous person care for the herd.* The tuberculous person is a source of greater danger to the herd than even the tuberculous animal. The latter can be confined to one place, and the virulent sputa can be prevented from drying and rising as dust, but for the attendant there is no such limitation, and his discharges are all the more dangerous. In one herd, where three of the family had died of tuberculosis, I found nineteen of the twenty-six cows badly affected.

By attending to such precautions, by killing all tuberculous animals and safely disposing of the carcasses, and by thoroughly disinfecting all the products, the stockowner may reasonably hope to purify his herd of the infection and to keep it sound.

I have purposely omitted all reference to methods of treatment, since these, however good for a patient whose life must be spared, necessarily entail a long-continued treatment, and are attended by too great expense and uncertainty in result to warrant their recommendation for animals when the object is to stamp out the disease.

### *Sanitary Police for Tuberculosis in Herds.*

In considering police methods for the extinction of tuberculosis in herds, our past experience in dealing with plagues of animals does not furnish an exact counterpart. The absolutely successful results in different parts of the world with rinderpest, lung-plague, sheep-pox, rabies, and glanders were obtained with diseases which confined their ravages to the lower animals, or which in attacking man entailed an acute and fatal disorder, so that the agency of man in propagating the contagium could be practically eliminated. In tuberculosis, on the other hand, we confront a disease which even in man often follows a tardy and occult course, and which is at all events habitually chronic, so that the contagium must be preserved so long as the phthisical man is allowed to go at large. It is not, however, so diffusive as the contagia of rinderpest, lung-plague, or sheep-pox, and hence the same absolute seclusion is not necessary to prevent its propagation.

If we could exclude dairy-herds from cities and dense aggregations of humanity, and see to it that consumptive persons were debarred from caring for or mingling with cattle and other susceptible animals elsewhere, we could proceed with the extinction of bovine tuberculosis with the same confident expectation of success as we have in the past undertaken the extinction of the diseases of animals just referred to. So long, however, as such separation is unattainable we must be content to be satisfied with less perfect results, and must be ready to repeat the process of extinction in the same herd as often as it may be necessary. Even with this drawback, we can promise a practical extinction of the affection in our herds and a reasonable guarantee of the soundness of our dairy and butcher products.

*Speedy Extinction of Tuberculosis in Herds.* In this direction I cannot do better than to repeat my suggestion in the Report of the Tuberculosis Commission for 1894. "All herds of the State would be examined and tested with tuberculin as speedily as possible; the diseased animals would be condemned, appraised, killed, and safely disposed of; the premises disinfected; other genera of animals that have lived with the diseased would be examined, and, if necessary, safely disposed of; vermin would be killed, and all consumptive persons would be *advised*" (enjoined) "against attending on the purified herd or preparing their food. Finally, all new purchases would be kept apart from the herd until they had been treated with tuberculin. In this way every step would be so much clear gain, and what had been once accomplished could be looked upon with resonable confidence as a permanent success." The continued testing of new purchases and the repeated examination of the herds at long intervals would be required, as isolated cases would at first appear from bacilli which had lodged on the respiratory or digestive mucosæ, but had not yet colonized the tissues prior to the tuberculin-test, and also from other accidental outside sources. Necropsies of all animals dying or killed would also be requisite so as to ensure that no seeds of the disease should be overlooked in any herd, to break out later.

The most serious objection to this prompt and effectual method is the lack of a sufficient appropriation. For indemnity alone, at $25 per head, 4 per cent. of our 2,500,000 of cattle (100,000) would demand $2,500,000. If we add to this the cost of inspection of the whole bovine race in the State, and of animals kept with them, of appraisements, and of the administration generally, we could not calculate on less than $3,000,000 as a working fund. Another drawback is the lack of professional men sufficiently acquainted with the use of tuberculin and with the diseases of animals generally to fill the rôle of inspectors satisfactorily. It would take a length of time to organize a satisfactory sanitary force, for the entire State, so that even if the money were available the inauguration of the enterprise would be necessarily slow.

*Registration of herds and necropsies to trace tuberculosis.* A most effectual but less speedy method of dealing with tuberculosis in herds would be based upon a universal registration of the bovine animals. To each county or district would be assigned one or more trustworthy veterinarians, who should keep an accurate record of animals, of all additions and removals, and who should examine all cattle killed or that died. On the discovery of a case of tuberculosis, the disease would be identified by the chief veterinarian, and the whole herd would be subjected to the tuberculin-test, and those found tuberculous would be condemned, appraised, killed, and safely disposed of, and the premises disinfected. Similar precautions as under the speedy method would be adopted toward other domestic animals on the same place, toward vermin, and toward tuberculous attendants and new purchases.

In this way every tuberculous herd would be discovered and purified without serious delay, and at the same time the expensive tuberculin-test would be reserved for herds in which tuberculosis was known to exist. The unfounded stock objection to the tuberculin-test, that it might introduce disease, would in this way be rendered absurd, even to the objector himself. This work could probably be accomplished by one hundred inspectors at a cost of $150,000 a year. Indemnities would also be lessened, as many of the occult cases would be detected only at the butcher's, and at least the demand for values would be more gradual and more easily met.

*Abattoir inspections to trace tuberculosis.* A third method of tracing tuberculosis is to provide municipal slaughter-houses in which alone farm-animals destined to be marketed as human food can be killed. This has long been the custom in Europe, and must one day be adopted in America as a purely sanitary measure, no matter how it may interfere with the vested interests and monopolies of individuals. Inspections in private abattoirs can never be made as satisfactorily as in public institutions where everything is done according to rule. Incidentally the government stamp and indorsement furnished with all sound meats would open to all competitors that interstate and foreign trade which is now the monopoly of the packers in certain large cities. Its effect in tracing the centres of animal tuberculosis one may judge from the reports of Berlin abattoirs, where 75 per cent. of old cows are tuberculous; of Leipsic, where 28 per cent. proved affected; of Schwerin, where 35 per cent. are diseased; of Paris, London, and Edinburgh, where 22 per cent. proved consumptive. With such data it is easy to follow each animal back to its herd and subject that to the tuberculin-test. If we were to combine this restriction of slaughter to inspected government abattoirs with a careful record of the herds and individual animals, it would prove easy and certain to discover every centre of disease and speedily to eradicate the contagion.

The great desideratum in our present methods is a definite and effective system. Our tuberculosis commissions are, in a sense, free lances, with power to rove about the State and test a herd here and a herd there, as the urgency of the demand and their own judgment may dictate. And in the preliminary inquiry as to the existence and relative prevalence of tuberculosis in different districts this has served a good purpose. But now that this purpose has been accomplished this should give place to a system which will look toward something more definite in the way of the extinction of the disease. Where a herd or district is purified of the infection due precautions should be taken to prevent its reinfection, so that the same work in such district will not be demanded again and again, and, above all, that the purified herd shall not be left exposed to diseased herds on all sides of it. Let herds be excluded from cities and suburbs, or, if that cannot be done, let the herds in such localities, the most dangerous to themselves and to humanity, be the first to be put under a rigid system for the suppression of the contagium. Next to these the dairy counties where the bovine population is the densest should be systematically attacked, and, finally, when these shall have been purified, the herds of the State at large, where the danger of the concentration of infection is least, should be taken in hand. Every step, however, should be subordinated to the maintaining of the ground that has already been gained, and the advance of the work in such lines as will render it possible or easy to hold all newly acquired territory. The general plan must, therefore, yield in many cases so as to clean up a whole district, the less densely peopled with cattle, as well as the more densely populated, so that the conquered territory may be kept compact and easily defensible from first to last. The time for the sporadic dealing with individual herds, removed from each other, is past. The knowledge and the needs of to-day unite in demanding such an appropriation and such supervision as will secure a systematic and progressive advance over the whole State, and which will look toward the purifying of our herds in every locality.

